# Basal and pentagastrin-stimulated calcitonin cut-off values in diagnosis of preoperative medullary thyroid cancer

**DOI:** 10.3906/sag-2003-182

**Published:** 2021-04-30

**Authors:** Emine KARTAL BAYKAN1, Mehmet ERDOĞAN

**Affiliations:** 1 Department of Endocrinology and Metabolism, Erzurum Regional Education and Research Hospital, Erzurum Turkey; 2 Department of Endocrinology and Metabolism, Ege University Medical Faculty, İzmir Turkey

**Keywords:** Medullary thyroid cancer, C cell hyperplasia, calcitonin, pentagastrin, cut-off

## Abstract

**Background/aim:**

Medullary thyroid cancer (MTC) originates from parafollicular cells (C cell) and produces calcitonin (CT). Basal serum CT was used in the diagnosis and treatment of MTC. If basal CT level is 100 pg/mL or higher, it is likely to have MTC, but if basal CT level is below 10 pg/mL, the probability of developing thyroid disease is low. In cases with basal CT level between 10–100 pg/mL, pentagastrin-stimulated (PS) CT level is studied to evaluate MTC and C cell hyperplasia (CHH). This study aimed to determine cut-off value for basal and PS peak CT level for diagnosis of MTC.

**Materials and methods:**

We retrospectively reviewed files of patients presented to endocrine outpatient clinic of Ege University, Medicine School, between 2010 and 2019; 176 patients with basal CT level of 10–100 pg/mL and patients with PS test were included to the study.

**Results:**

The receiver operating characteristic curve (ROC) analysis was used to determine cut-off value for basal CT that can discriminate cases with MTC and those with nodular goiter. Cut-off value for basal CT was calculated as 46.5 pg/mL (specificity; 100 %, sensitivity; 74 %). In the ROC analysis for peak PS CT, cut-off value was calculated as 285 pg/mL (specificity:100 %; sensitivity:82 %). When peak CT level was > 290 pg/mL in PS test, both specificity and sensitivity for MTC were determined as 100 %. The PS peak CT level > 285 pg/mL was significant for MTC diagnosis while range of 117–274 pg/mL was significant for CHH.

**Conclusion:**

In this study, cut-off value was calculated as 46.5 pg/mL for basal CT, whereas 285 pg/mL for PS peak CT in the diagnosis of preoperative MTC.

## 1. Introduction

The medullary thyroid cancer arises from parafollicular C cells and produces calcitonin (CT). It is a rare cancer accounting for 4%–10% of all thyroid cancers [1]. The MTC is sporadic in 75% while familial in 25 % of cases [2,3]pheochromocytomas, mucosal neuromas, ganglioneuromas of the intestinal tract, and skeletal and ophthalmic abnormalities. It appears both as an inherited disorder and as de novo disease. Sequence analysis of germ-line DNA from MEN 2B patients revealed the existence of the same point mutation in the RET protooncogene in 34 unrelated individuals. This sequence difference was not observed in 93 unaffected individuals, including the normal parents of 14 de novo MEN 2B patients. The mutation (ATG → ACG. The major factor predicting morbidity and mortality is early diagnosis and radical surgery in MTC. The MTC is a highly aggressive malignancy with a 10-years life expectancy of 40%–50% [4,5]using uni- and multivariate analysis. DESIGN AND PATIENTS. Clinical, biological, surgical and epidemiological data on 899 MTC patients, diagnosed between 1952 and 1996, were collected by the French Calcitonin Tumors Study Group (GETC. 

Basal serum CT level is used in the diagnosis and follow-up of MTC. The CT is a polypeptide involving 32 amino acids, which is produced by parafollicular C cells of thyroid. If basal CT level is 100 pg/mL or higher, it is likely to have MTC. On the other hand, if basal CT level is below 10 pg/mL, the probability of developing thyroid disease is low [6]. An elevated level of serum calcitonin is a highly sensitive marker for MTC, but it is not especially specific. Also, some studies reported that 10% to 40% of all patients with thyroid nodules associated with high basal levels of calcitonin had MTC [7]calcitonin measurements are mostly useful in the evaluation of tumor size and progression, and as an index of biochemical improvement of medullary thyroid carcinomas. Although measurement of calcitonin is a highly sensitive method for the detection of medullary thyroid carcinoma, it presents a low specificity for this tumor. Several physiologic and pathologic conditions other than medullary thyroid carcinoma have been associated with increased levels of calcitonin. Several cases of thyroid nodules associated with increased values of calcitonin are not medullary thyroid carcinomas, but rather are related to other conditions, such as hypercalcemias, hypergastrinemias, neuroendocrine tumors, renal insufficiency, papillary and follicular thyroid carcinomas, and goiter. Furthermore, prolonged treatment with omeprazole (> 2-4 months.  In cases with basal CT level between 10 and 100 pg/mL, pentagastrin-stimulated CT level is studied to evaluate MTC and C cell hyperplasia (CHH). The pentagastrin-stimulated CT level is highly sensitive in demonstrating C cell disease [8]. There is no definitive data regarding cut-off value for CT level after pentagastrin stimulation test. This study aimed to determine cut-off value for basal and pentagastrin-stimulated peak CT level for diagnosis of MTC.

## 2. Materials and methods

The protocol was conducted in accordance with the Declaration of Helsinki and approved by the Ethics Committee (2020/06-73). All participants were informed about the survey.

We retrospectively reviewed files of patients presented to endocrine outpatient clinic of Ege University, Medicine School Overall, between 2010 and 2019; 176 patients with basal CT level of 10-100 pg/mL and pentagastrin stimulation test were included to the study. 

### 2.1. Pentagastrin stimulation tests 

All 176 patients underwent calcitonin stimulation testing first with pentagastrin. Pentagastrin was administered as an intravenous(iv) injection of 0.5 μg/kg body weight over 10 s of iv infusion. Blood samples were drawn before the application of the stimulant as well as, 1, 2, 3, 5, and 10 min after and were used for the measurement of calcitonin. Peak calcitonin level was considered significant.

Patients with secondary causes (renal failure, proton-pump inhibitor use, atrophic gastritis, Hashimoto’s thyroiditis, pseudohypoparathyroidism, hepatic cirrhosis, and hypergastrinemia) of CT elevation were excluded. All patients had undergone thyroid surgery, and postoperative histopathology results were available.

### 2.2. Calcitonin assay

CT assay was performed using IMMUNITE 1000 chemiluminescence analyzer (Siemens AG, Munich, Germany). Detection limit is 2 to 2,000 pg/mL. Blood samples were drawn basally 1, 2, 3, 5 and 10 min after pentagastrin injection. 

### 2.3. Statistical analysis

We performed all statistical analyses using SPSS for Windows (Version 17.0. Chicago, IL, USA). Unless otherwise stated, results were expressed as mean ± SD. We used the Mann–Whitney U test or independent sample t test between two subject groups, and used the Pearson correlation test or Spearman correlation test, as appropriate. Comparison of multiple groups was made by performing an analysis of variance with Bonferroni correction. Comparison of pretest and posttest results was made as dependent sample test analysis and Wilcoxon signed rank test. The ROC analysis was used to determine whether a continuous variable can be used in the diagnosis. Results are presented with 95% confidence interval. The P value < 0.05 was considered as statistically significant. 

## 3.Results

Based on postoperative histopathology results, there was benign nodular goiter in 122 patients, MTC in 23 patients, CHH in 12 patients and nonMTC thyroid cancer in 19 patients. The patients were stratified into 4 groups. 

Of 176 patients included, 84 were women, whereas 92 were men. When sex was assessed among groups, the percentage of patients with female sex was higher in patients with MTC, but no statistically significant difference was found when compared with other groups (Chi-square test P: 0.059). In the other three groups, the percentage of male sex patients was higher (Table 1). 

**Table 1:** Data of groups (*P < 0.05).

	Age	N	Calcitonin	Pentagastrin stimulation test
BenignFemaleMale	49.8	12259 (%48.4)63 (%51.6)	14.53 ± 6.13 pg/mL16.4 ± 8.7 pg/mL13.6 ± 4.3 pg/mL	46.77 ± 34.14 pg/mL43 ± 28.5 pg/mL48 ± 36.7 pg/mL
MTCFemaleMale	49.4	2313 (%56.5)10 (%43.5)	39.93 ± 28.53 pg/mL* 42 ± 27.6 pg/mL 46 ± 32.8 pg/mL	459.10 ± 494.37 pg/mL *485 ± 512 pg/mL 560 ± 676 pg/mL
CHHFemaleMale	43*	125 (%41.7)7 (%58.3)	14.57 ± 3.3 pg/mL 14.6 ± 3.2 pg/mL14.5 ± 4.94 pg/mL	195 ± 51.39 pg/mL193 ± 61 pg/mL198 ± 24 pg/mL
NonMTC thyroid cancerFemale Male	50.9	197 (%36.8)12 (%63.2)	15.79 ± 4.14 pg/mL13.7 ± 5.1 pg/mL17.1 ± 3.9 pg/mL	67.36 ± 47.04 pg/mL60 ± 45 pg/mL70 ± 51 pg/mL

Mean age was 51.79 ± 12.66 (range: 24–83 years). The mean age was significantly lower in CHH Group when compared to remaining groups (p: 0.033) where mean age was comparable but the number of patients in this group was 12 and there were less patients than the other groups (Table 1).

**Calcitonin:** Mean CT level was 14.53 ± 6.13 (10–43 pg/mL) in benign nodular goiter group, 39.93 ± 28.53 (10–85 pg/mL) in the MTC group, 14.57 ± 3.3 pg/mL (11-18 pg/mL) in CHH and 15.79 ± 4.14 pg/mL (10–23 pg/mL) in nonMTC thyroid cancer group. 

Basal CT levels of four groups were compared with bonferroni correction and analysis of variance. It was found that basal CT level was significantly higher in MTC group than remaining groups (P: 0.000). There was no significant difference in basal CT level among CHH, benign nodular goiter and nonMTC thyroid cancer groups (Table 1). 

Cut-off value for basal CT was calculated as 46.5 pg/mL with specificity of 100 % and sensitivity of 74 % for MTC diagnosis (Area under Curve [AUC]: 0.720; 95 % CI: 0.027–0.117; P: 0.000). The cut-off level for basal CT was calculated as 46.5 pg/mL in women (sensitivity: 68 %; specificity: 100) (AUC: 0.944; 95 % CI: 0.09–0.103; P: 0.000) and 53 pg/mL in men (sensitivity: 84 %; specificity: 100 %) (AUC: 0.935; 95 %: 0.875–0.995; P: 0.001) (Table 1).

When basal CT level was linked to MTC diagnosis, it was found that MTC was present by 100% when CT level was above 100 pg/mL (Table 2). 

**Table 2:** The relationship between CT level and MTC diagnosis.

Basal calcitonin	MTC diagnosis
< 10 pg/mL	0%
10-15 pg/mL	19.1 %
15-20 pg/mL	25.6 %
20-50 pg/mL	40.5 %
50-100 pg/mL	88.5 %
≥ 100 pg/mL	100 %

**Pentagastrin stimulation test:** The mean peak pentagastrin-stimulated CT level was 46.77 ± 34.14 pg/mL (10-142 pg/mL) in benign nodular goiter group, 459.10 ± 494.37 pg/mL (108 - 1938 pg/mL) in MTC group, 195 ± 51.39 pg/mL (117 - 274 pg/mL) in CHH group and 67.36 ± 47.04 pg/mL (14 - 149 pg/mL) in non-MTC thyroid cancer group. 

When pentagastrin-stimulated peak CT levels were compared (bonferroni correction and analysis of variance) among groups, it was found to be significantly higher in MTC group (108 –1938 pg/mL) than remaining groups (p: 0.000). Pentagastrin-stimulated peak CT level ranged from 117 to 274 pg/mL in CHH group, indicating significant difference between CHH and benign nodular goiter and nonMTC thyroid cancer groups (P: 0.000).

When the dependent sample test was analyzed in the Wilcoxon signed-rank test to compare the results of all groups before and after PG stimulation, the difference was statistically significant. (P: 0.000).

 In the ROC analysis for peak pentagastrin-stimulated CT, cut-off value was calculated as 285 pg/mL (specificity: 100 %; sensitivity: 82 %; AUC: 0.928; 95% CI: 0.0–0.26; P: 0.000). When ROC analysis was performed between MTC and CHH groups, cut-off value for peak CT was calculated as 285 pg/mL (specificity: 100 %; sensitivity: 93.8 %; AUC: 0.948; 95 % CI: 0.0–0.26; P: 0.000). When peak CT level was > 290 pg/mL in pentagastrin stimulation test, both specificity and sensitivity for MTC were determined as 100%.

When the correlation between the peak CT level and MTC was evaluated, the MTC was present at 100% when the peak CT level was> 290 pg / mL (Table 3).

**Table 3:** The relationship between PG stimulated CT level and MTC diagnosis.

Peak Calcitonin	MTC diagnosis
< 100 pg/mL	0 %
100–150 pg/mL	41.66 %
150–250 pg/mL	70 %
250–275 pg/mL	84.6 %
275–290 pg/mL	93.8 %
> 290 pg/mL	100 %

## 4. Discussion

In this study, we determined the basal cut-off value and pentagastrin-stimulated peak CT level for the diagnosis of MTC. Cut-off value for basal CT was calculated as 46.5 pg/mL with specificity of 100 % and sensitivity of 74 %. Cut-off value for pentagastrin-stimulated peak CT level was found to be 285 pg/mL in the diagnosis of MTC. Peak CT level was found to be < 274 pg/mL in cases with CHH.

Medullary thyroid cancer (MTC) is the third most common thyroid malignancy. It originates from the parafollicular or calcitonin-producing C cells and maintains the features of these cells [9,10], occurs in 3%–10% of cases of thyroid cancer [11]whereas no unequivocal rise was found after the age of 20 for familial disease. Standardized morbidity ratios (SMR. MTC can be sporadic or familial, in 75% and 25% of cases [12].

The prognosis of MTC is unfavorable, with a 10-year survival rate of MTC patients of approximately 50%. The cure and survival rates of these patients are positively affected by an early diagnosis and precocious surgical treatment [13,14]who had had their primary surgical treatment at the Mayo Clinic during the years 1946 through 1970. Of these patients, 58 had sporadic and 7 had familial medullary thyroid carcinoma. Thyroid nodules were the most common initial manifestation. Near-total thyroidectomy was the most frequent initial operation. Survival was affected by the following factors: male sex, familial inheritance, size of the tumor, stage of the tumor (American Joint Committee on Cancer.

The most common presentation of sporadic cases is thyroid nodular disease. Serum calcitonin is the most sensitive biochemical marker for both the primary diagnosis of MTC and for follow-up. At variance, elevated basal levels of serum calcitonin, especially when greater than 100 pg/mL, are diagnostic of MTC. Routine measurement of serum calcitonin in nodular thyroid diseases provides preoperative diagnosis of sporadic MTC [15–19]mean age 42.8 years, range with 18-76 years. Patients diagnosed with serum calcitonin screening have been shown to have significantly better prognosis than those diagnosed with cytology or histology [18]we demonstrated that routine measurement of serum calcitonin (CT. Among our patients, benign cytology was detected in the preoperative fine needle biopsies (FNAB) in the group with benign thyroid nodules. Measurement of basal calcitonin levels in patients with malignant or suspicious FNAB may be a cost-effective approach to screen for MTC. There are several other conditions, however, in which basal levels of serum calcitonin may be elevated. Hypercalcitoninemia may be observed in nonMTC conditions including autoimmune thyroid disease, chronic kidney disease, use of proton pump inhibitors, hypergastrinemias, the presence of heterophilic antibodies, and nonthyroidal neuroendocrine tumours of the foregut, pancreas, prostate, and lung [20–26]the median basal CT concentration of 17 patients was 31 pg/mL (13-76 

Calcitonin is the most sensitive tumor marker for MTC and its measurement is used to monitor patients with residual and metastatic MTC [27]. However, CT is not specific for MTC and has no established cut-off value. The sensitivity and the specificity of basal serum CT assays can be improved stimulated CT testing. Stimulated CT testing was historically done by measuring serum CT levels after intravenous administration of calcium and/or pentagastrin. The pentagastrin stimulation test has been used widely for many years to stimulate calcitonin.

 In this study, we determined the basal cut-off value for the diagnosis of MTC. The ROC analysis was used to determine cut-off value for basal CT that can discriminate cases with MTC and those with nodular goiter. Cut-off value for basal CT was calculated as 46.5 pg/mL with specificity of 100% and sensitivity of 74%. Both specificity and sensitivity for basal CT > 100 pg/mL were 100%. The cut-off level for basal CT was calculated as 46.5 pg/mL, in women and 53 pg/mL in men. 

CT levels are slightly lower in women than in men [28,29] due to the sex dependent differences in lower basal CT levels and to the fact that men have a larger C-cell mass than women [30,31]CT measurement can vary with assay- and nonassay-dependent factors, and procalcitonin (PCT. 

There is limited number of studies which determined cut-off value for basal CT level, reporting different cut-off values ( ≥ 68 pg/mL, ≥ 46 pg/mL and ≥ 32 pg/mL for men and ≥ 35 pg/mL, ≥ 26 ng/mL and ≥ 18 pg/mL for women) [32–37]RET gene mutations carriers (n = 14.

The cut-off level of CT to initiate pentagastrin testing remains controversial because basal CT values between 10 and 100 represent a gray zone where true- and false-positive cases overlap [38–42]the prevalence of MTC was 1.37% and reached 41.1% when the basal CT level was abnormal (3% of the patients. We have performed pentagastrin testing in all patients with CT > 10  pg/mL without losing any of these patients to follow-up. 

In this study, we determined cut-off value for pentagastrin-stimulated peak CT level for diagnosis of MTC. In the ROC analysis, cut-off value for pentagastrin-stimulated peak CT level was found to be 285 pg/mL in the diagnosis of MTC. Peak CT level was found to be < 274 pg/mL in cases with CHH. In the literature, there is limited number of studies reporting cut-off value for peak CT level for diagnosis of MTC and peak CT level that discriminates MTC from CHH. Scheuba et al. reported pentagastrin-stimulated peak CT level as 560 ng/mL in the diagnosis of MTC, whereas < 129 pg/mL in CHH [41]. Milone et al. found pentagastrin-stimulated peak CT level ass 275 pg/mL for diagnosis of MTC [43]. 

However, the risk of MTC is very low in the range of 10–15 pg/mL basal CT and CT < 100 pg/mL after pentagastrin stimulation. This fits the upper limit of the normal 100 pg/mL range, as published in the 2004 German consensus proposal. Citation?? Annual follow-up research recommended in patients with pentagastrin stimulation of aCT between 50 and 100 pg/mL shed new light on this issue. Lowering this threshold for surgery can lead many patients to be directed to unnecessary operations. Therefore, the identification of 100 pg/mL as the therapeutic threshold reflects a clinical agreement that balances overtreatment and undertreatment in these patients [44].

Limitations of our study were retrospective design, absence of data of FNAB results for all patients, absence the data of smoking and body mass index data.

In conclusion, cut-off value was found as 46.5 pg/mL for basal CT level and 285 pg/mL for pentagastrin-stimulated peak CT level in the preoperative diagnosis of MTC. The pentagastrin-stimulated peak CT level > 285 pg/mL was significant for MTC diagnosis, while level of 117-274 pg/mL was significant for CHH. 

## Informed consent

Ethics committee approval was received for this study from the Ethics Committee (2020/06-73). Written informed consent was obtained from the patients who participated in this study.
